# Impact of Microkeratome Dissection Parameters on Textural Interface Opacities in DSAEK Grafts

**DOI:** 10.3390/diagnostics15131608

**Published:** 2025-06-25

**Authors:** Marina S. Chatzea, George D. Kymionis, Dionysios G. Vakalopoulos, Robert C. O’Brien, Daniella Mora, Katrina Llanes, Elizabeth Fout, William Buras, Concetta Triglia, Rahul S. Tonk, Sonia H. Yoo

**Affiliations:** 1 1st Department of Ophthalmology, “G. Gennimatas” Hospital, National and Kapodistrian University of Athens, 11527 Athens, Greece; marinachatzea@gmail.com (M.S.C.); dionisis.vakalopoulos@gmail.com (D.G.V.); robrien@med.miami.edu (R.C.O.); 2 Miller School of Medicine, University of Miami, Miami, FL 33136, USA; 3 Beauty of Sight, Bascom Palmer Eye Bank, Miami, FL 33136, USA; da651115@ucf.edu (D.M.); knl39@med.miami.edu (K.L.); efcaraza@med.miami.edu (E.F.); wbb25@med.miami.edu (W.B.); ctriglia@med.miami.edu (C.T.); 4 Corneal and External Diseases, Bascom Palmer Eye Institute, School of Medicine, University of Miami Miller, Miami, FL 33136, USA; rtonk@med.miami.edu (R.S.T.); syoo@med.miami.edu (S.H.Y.)

**Keywords:** DSAEK, interface, opacities, TIO, microkeratome

## Abstract

**Background**: Textural interface opacities (TIOs) following Descemet’s stripping automated endothelial keratoplasty (DSAEK) have become a significant postoperative concern. Studies have explored possible links such as stromal irregularities and viscoelastic usage, but the exact cause of TIOs remains unclear. PURPOSE: To evaluate the relationship between microkeratome dissection parameters and the development of textural interface opacities in DSAEK grafts utilizing the “M-TIO” grading scale for standardized assessment. **Methods**: Optical coherence tomography (OCT) images of DSAEK-processed corneal grafts, prepared with the same microkeratome and technique for transplantation at Bascom Palmer Eye Institute, underwent blinded analysis using a newly developed grading scale termed “M-TIO”. This analysis aimed to evaluate and categorize the occurrence of TIO, explore its potential correlation with graft characteristics prior to DSAEK preparation, and assess specific stages of the DSAEK dissection process. Data collected included the size of the microkeratome head used, the difference between the head and the actual stromal cut, and the difference between the pre-cut graft thickness and post-cut DSAEK lenticule thickness. **Results**: The study retrospectively included 422 donor corneas transplanted from 2019 to 2023. Variables associated with TIO in the final multivariable ordinal logistic model included the difference between the pre-cut graft thickness and the post-cut DSAEK lenticule thickness (OR: 1.57 [99% CI: 1.22 to 2.06] per 50 µm) and microkeratome head (OR: 6.95 [99% CI: 1.04 to 36.60] 300 µm, OR: 4.39 [99% CI: 0.76 to 19.00] 350 µm, and OR: 18.86 [99% CI: 2.35 to 175.91] 400 µm vs 450 or 500 µm, respectively). **Conclusions**: This study identified a statistically significant association between TIOs and the microkeratome DSAEK preparation, proposing several factors that could help prevent its occurrence. Specifically, creating an ultra-thin DSAEK lenticule from an initially thick graft using a smaller microkeratome head with the slow single-pass technique may increase the risk of TIOs. In contrast, utilizing a larger microkeratome head can improve stromal thickness consistency, reduce technical challenges during graft preparation, and lower the risk of TIOs.

## 1. Introduction

**Figure 1:**
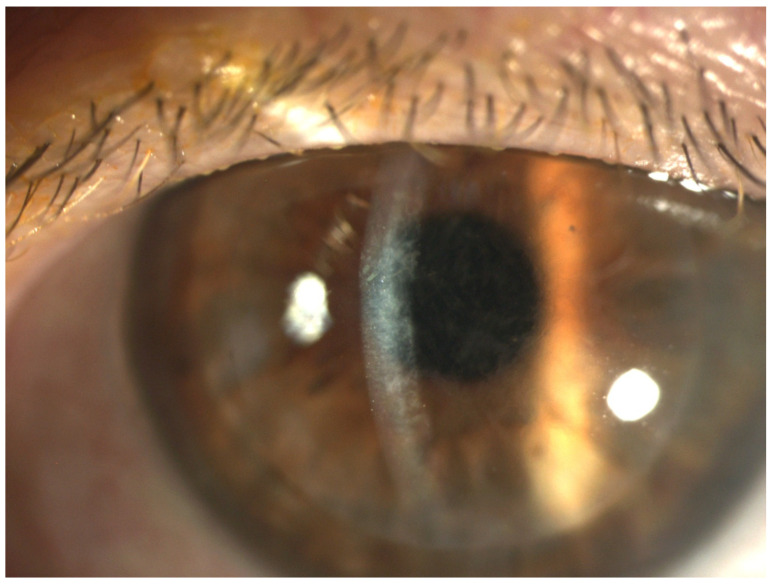
Slit-lamp photograph following DSAEK depicting the diffuse TIO.

Endothelial keratoplasty (EK) is currently the standard surgical procedure for managing endothelial dysfunction when no corneal scarring is present. According to the Eye Bank Association of America’s statistical report, in 2023, Descemet membrane endothelial keratoplasty (DMEK) became the most commonly performed keratoplasty procedure in the United States, totaling 17,116 cases. Nevertheless, Descemet stripping automated endothelial keratoplasty (DSAEK) remained closely prevalent, accounting for 16,207 procedures, underscoring its ongoing clinical importance [1]. Ultrathin Descemet stripping automated endothelial keratoplasty (UT-DSAEK) has demonstrated significantly improved visual outcomes compared to standard DSAEK, achieving results comparable to DMEK while maintaining the low complication rates associated with conventional DSAEK [2,3,4,5,6]. Compared to penetrating keratoplasty (PK), which accounted for 14,486 procedures [1]. DSAEK offers faster visual recovery, improved refractive results, increased tectonic stability, and a lower risk of suture-related infections [7]. While DSAEK remains a well-established and commonly performed technique, it is associated with several donor-recipient interface complications, such as infections, epithelial ingrowth, retained Descemet membrane, interface folds, and stromal deposits [8,9]. Among these, textural interface opacities (TIOs)—also referred to as “interface wave-like deposits,” “reticular haze,” or “ground glass interface haze” in the literature—have gained recognition as a significant postoperative issue. The visual impact of TIOs varies widely, ranging from severe forms requiring surgical management [10] to mild cases that gradually regress without impacting vision [11]. These opacities are defined by the presence of inert material accumulating at the graft–host interface, typically appearing as diffuse, central, and often undulating grayish haze detectable on slit-lamp examination (Figure 1). Although several studies have attempted to identify the exact cause [8,12], the underlying mechanism seems to involve a combination of the donor stroma irregularity and the amount of viscoelastic material trapped within the irregular fibers [8,12,13]. TIOs, while relatively rare, represent a significant postoperative complication that can compromise visual acuity and, in persistent or severe cases, may necessitate additional surgical intervention [10,11].

TIOs were first identified as a postoperative complication associated with laser in situ keratomileusis (LASIK) [14,15,16,17]. A common factor between LASIK and DSAEK is the creation of stromal cuts, typically performed with either a microkeratome or a femtosecond laser [17,18]. These stromal cuts could be the cause of irregular surface fibers, which have been recognized as a significant contributor to the development of TIOs [13,17].

Femtosecond laser dissection is generally thought to create more irregular stromal surfaces than mechanical microkeratome dissection [17,18]. This can be explained by the laser’s mechanism of tissue separation. Femtosecond lasers operate through photodisruption, a process in which tightly spaced cavitation bubbles are created within the stroma to cleave the tissue. Unlike the continuous, smooth pass of a mechanical blade, this technique relies on a sequence of micro-explosions to achieve dissection [18,19]. As a result, small gaps between the bubbles and residual stromal fibers often remain, leading to a more irregular and textured stromal surface. Several studies have demonstrated that this textural interface is associated with increased early postoperative backscatter and interface haze in femtosecond-assisted LASIK compared to microkeratome-assisted procedures [15,19]. While these irregularities typically resolve over time and have minimal impact on long-term visual outcomes, due to the limited extent and transient nature of the residual fibers, the evidence highlights the influence of dissection technique on interface quality [15]. 

Although the mechanism of interface irregularity in femtosecond laser dissection is well characterized, the underlying cause of similar stromal surface irregularities occasionally observed after microkeratome dissection remains unclear. This study aims to investigate the potential association between TIOs and the microkeratome-cutting process used in the DSAEK preparation of corneal grafts.

## 2. Materials and Methods

### 2.1. DSAEK Preparation

This retrospective, single-center study was conducted over five years, from January 2019 through December 2023. It involved comprehensive analysis of optical coherence tomography (OCT) imaging from donor corneal grafts prepared for DSAEK. All donor tissues were processed at the Beauty of Sight, Florida Lions Eye Bank, following a uniform protocol using a rotational microkeratome system (Moria ALTK CBm Combo system; Moria SA, Antony, France). Initially, donor corneas were stored under hypothermic conditions between 2–8 °C, consistent with established standards to maintain endothelial cell viability. Before lamellar dissection, each cornea was allowed to equilibrate to room temperature, ensuring consistent biomechanical properties and enhancing microkeratome cutting accuracy. Tissues were mounted on an artificial anterior chamber (AAC system, Moria SA), and chamber pressure was adjusted to provide optimal corneal rigidity and surface stability during dissection. Central corneal thickness was measured via anterior segment OCT both before and after the microkeratome pass. The appropriate microkeratome head size was chosen based on initial corneal thickness measurements and target graft thickness. A standardized slow single-pass dissection method was applied for all graft preparations to improve consistency and minimize variability in graft thickness [6,20,21].

### 2.2. Data Analysis

**Figure 2:**
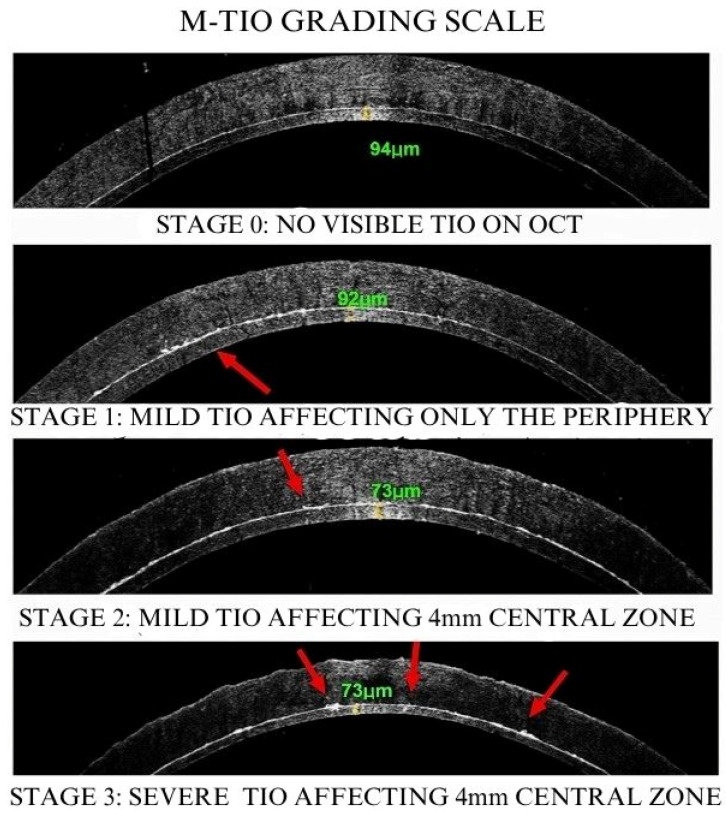
“M-TIO” Grading Scale of DSAEK grafts assessing TIO prior to transplantation. Red arrows indicate irregular hyperreflective areas representing TIOs in the graft interface.

Based on prior research indicating that TIO manifests as hyperreflectivity on the donor surface in OCT images [9,12], a four-stage “M-TIO” [22] grading scale was established to classify TIOs based on the extent of hyperreflectivity (Figure 2). In Stage 0, there is no visible TIO on OCT. Stage 1 involves mild TIO that affects only the periphery of the cornea, specifically beyond the central 4 mm zone. In Stage 2, mild TIO affects the central zone of the cornea, defined as the 4 mm area that represents the average mesopic pupil of adults older than 65 years old [23]. Finally, Stage 3 is characterized by severe TIO affecting the central zone, presenting as a thicker hyperreflective area. All OCT images of DSAEK-processed corneal grafts were evaluated in a blinded manner by three independent corneal specialists to assess the significance of TIO in preoperative evaluations. 

Following this assessment, the visual acuity outcomes of patients who received the corresponding DSAEK grafts were recorded at the 1-year postoperative evaluation. All procedures were performed using a standardized surgical technique by expert corneal specialists in accordance with the standard of care at Bascom Palmer Eye Institute. Data collection included clinical profiles of the patients, imaging findings, management strategies, and final visual and clinical outcomes. Recipients who received ultra-thin DSAEK grafts < 100 μm, for the treatment of Fuchs corneal dystrophy were included in the study, while patients with ocular comorbidities, complicated DSAEK procedures, and DSAEK grafts > 100 μm [3,4,5,6], which could negatively impact visual outcomes, were excluded. This approach allowed us to control for variability by ensuring that all patients had comparable visual potential and received uniform surgical and postoperative care, thereby minimizing the likelihood that patient-related factors or differences in postoperative management influenced the outcomes.

In this study, we selected to evaluate pinhole visual acuity (PHVA) instead of best-corrected visual acuity (BCVA) as our primary outcome measure. This decision was based on the recognition that postoperative irregular astigmatism, which may result from factors including interface irregularities or healing responses, cannot always be fully corrected with spectacles or standard refractive methods. Additionally, visual disturbances like glare and halos [15,24,25], which could be associated with TIO, may persist despite optimal refractive correction and are not assessed by BCVA. PHVA effectively reduces the impact of refractive errors and higher-order aberrations, providing a more accurate representation of the potential visual acuity in the presence of such irregularities [26,27,28]. This approach aligns with clinical practices where pinhole testing is employed to differentiate between refractive and non-refractive causes of visual impairment, particularly in conditions involving corneal irregularities.

To evaluate the microkeratome DSAEK cutting process, we collected data including the pre-cut graft thickness, post-cut DSAEK lenticule thickness, the size of the microkeratome head used, and the Moria set employed for each graft. This information allowed us to calculate the difference between the pre-cut graft thickness and post-cut DSAEK lenticule thickness, as well as the discrepancy between the size of the blade and the actual stromal cut. Additionally, given previous research linking cataract surgery to biomechanical alterations in the cornea, we also documented the donor’s lens status to determine if possible stromal changes of the donor cornea would have any impact on the microkeratome cutting process.

The primary objective of this study was to evaluate donor-related factors and parameters associated with DSAEK lenticule preparation that may influence the development and severity of TIO in DSAEK grafts. To achieve this, we employed the “M-TIO” grading scale, which classifies TIOs based on the extent and location of hyperreflectivity observed in OCT imaging. Using this classification, we assessed whether specific parameters were associated with an increased incidence or severity of TIOs. By correlating each of these factors with the “M-TIO” stage assigned to the corresponding graft, we aimed to identify which variables were predictive of greater interface irregularity.

### 2.3. Statistical Analysis

Statistical analyses were performed using R version 4.4.2 with the boot, boot.pval, brant, brglm2, lmtest, MASS, performance, and tidyverse packages. Ordinal logistic regression was used to analyze various donor and eye bank variables as predictors of TIO grade, and proportional odds ratios (ORs) greater than 1 indicate greater odds of more severe TIO. The final multivariable ordinal logistic model was selected using all possible regressions with the Bayesian information criterion (BIC). A two-sided *p* value < 0.01 was considered statistically significant.

## 3. Results

From 2019 to 2023, there were 76 (18%), 224 (53%), 97 (23%), and 25 (6%) donor corneas with TIO grades of 0, 1, 2, and 3, respectively, according to “M-TIO” scale. Table 1 displays descriptive statistics (i.e., means and standard deviations) of pre-cut donor cornea graft thickness, post-cut DSAEK lenticule thickness, and their difference by “M-TIO” scale grade. Thicker pre-cut donor corneas were observed among higher M-TIO grades, thinner post-cut DSAEK lenticule grafts were observed among higher M-TIO grades, and greater differences between the pre-cut graft thickness and the post-cut DSAEK lenticule were observed among higher M-TIO grades.

The proportional odds assumption was satisfied for all models except the univariable model for donor lens status (*p* = 0.04). This finding suggests that donor lens status, particularly pseudophakia, does not have a statistically significant association with the development of TIOs. Although cataract surgery and the use of viscoelastic agents are known to alter corneal biomechanics, reflected in changes to parameters such as corneal hysteresis and corneal resistance factor [29], our results indicate that these biomechanical modifications are not directly implicated in the formation of TIO during DSAEK graft preparation. For all other variables, the assumption held with *p*-values ≥ 0.18.

Estimated proportional ORs from the univariable ordinal logistic regression models with each candidate variable predicting TIO are provided in Table 2. Statistically significant associations were observed between M-TIO grade and pre-cut donor cornea thickness, the difference in pre-cut graft thickness and the post-cut DSAEK lenticule thickness, microkeratome head size, dissection thickness vs. microkeratome head size, and year of donor cornea procurement (all *p*’s < 0.001). No other variables considered in this study were significantly associated with TIO (all *p*’s ≥ 0.08) (Table 2).

Variables associated with TIOs in the final multivariable ordinal logistic model, selected via BIC, included the difference pre-cut graft thickness and post-cut DSAEK lenticule thickness (OR: 1.57 [99% CI: 1.22 to 2.06] per 50 µm; *p* < 0.001) and microkeratome head (OR: 6.95 [99% CI: 1.04 to 36.60] 300 µm, OR: 4.39 [99% CI: 0.76 to 19.00] 350 µm, and OR: 18.86 [99% CI: 2.35 to 175.91] 400 µm vs 450 or 500 µm, respectively; *p* < 0.001). (Table 3).

## 4. Discussion

This study aimed to explore a possible cause of TIOs in DSAEK. Based on previous research linking TIO to irregular donor stroma, an OCT image-based grading scale was developed to assess stromal irregularity and investigate a possible association between TIO severity and the microkeratome-cutting process.

Our results indicate a significant association between pre-dissection donor cornea thickness and TIOs, where a thicker initial graft increased the likelihood of TIO occurrence. In contrast, DSAEK lenticule graft thickness showed a *p*-value of 0.08, suggesting a weak but statistically insignificant association between post-cut DSAEK lenticule thickness and TIO formation. However, the difference in pre-cut corneal graft thickness and post-cut DSAEK lenticule thickness was associated with TIOs, where a larger difference between initial and final graft thickness increased the likelihood of TIOs.

To better understand the results regarding the microkeratome head size and its difference from the actual stromal cut thickness, it is important to review the microkeratome technique used in the DSAEK process. Research supports that the slow single-pass microkeratome technique is both safe and effective for creating ultra-thin DSAEK grafts [2,6,20]. In the Moria ALTK CBm Combo rotated system (Moria SA, Antony, France), the largest available microkeratome head size is 400 μm. In this context, for edematous corneal grafts, which can exceed 550 μm in thickness, technicians must adjust the DSAEK process. In the single-pass technique, thicker grafts present greater resistance to the microkeratome as a result of the increased stromal volume. Additionally, to achieve a deeper cut, both the duration of the microkeratome pass and the pressure applied to the artificial anterior chamber must be increased, further contributing to the technical challenges of the procedure. These modifications can result in a dissection plane that extends beyond the nominal depth specified by the microkeratome head, often by approximately 100 μm, creating a significant difference between the microkeratome head size and the actual stromal cut, resulting in a thinner DSAEK lenticule. Our study identified this difference as a contributing factor to the higher likelihood of developing severe TIO, particularly in DSAEK-processed corneal grafts classified as stage 3 according to the grading scale. 

The observed correlation between TIOs and year may be attributed to the growing clinical demand for ultra-thin DSAEK lenticules, which led to more frequent use of the slow single-pass microkeratome technique to achieve grafts thinner than the blade’s nominal depth. In 2022, the largest available blade was 350 µm, often requiring a slower dissection to reach the desired graft thickness. In 2023, the introduction of a 400 µm blade enabled the preparation of thinner grafts while maintaining a more optimal dissection plane, which may have contributed to the reduced incidence of TIOs compared to 2022. However, we found that the use of the 400 μm microkeratome head was associated with a higher incidence of TIOs, likely due to technicians defaulting to the largest available size when attempting to create a very thin DSAEK lenticule from a thicker graft. Pre-cut donor corneas typically have a central thickness between 450 and 600 μm, while the target thickness for ultrathin DSAEK lenticules is <100 μm. When the donor cornea thickness is between 450 and 500 μm, the use of a 400 μm microkeratome head is generally appropriate and may result in a consistent, thin lenticule. However, in cases where the donor thickness exceeds 500 μm, the application of a 400 μm head, with a slow single-pass dissection technique, can lead to a significant disparity between the pre-cut graft thickness and post-cut DSAEK lenticule thickness, which has been shown to increase the incidence of TIO formation. Conversely, we demonstrated that using microkeratome heads of 450 and 500 μm with a quick, single microkeratome pass, available in the linear ALTK CBm Combo system, was associated with a lower likelihood of TIO formation. This suggests that greater head sizes and precise technique adjustments may help reduce the occurrence of severe TIO.

The slow single-pass technique has been preferred by many eye banks due to long-term practice experience and its cost-effectiveness compared to the linear microkeratome models, which necessitate a single-use head for each DSAEK graft preparation procedure. Although this technique is well established and widely adopted, our study indicates that the induced vibrations during the slow single-pass process may contribute to irregularities in the donor stroma, thereby increasing the likelihood of developing TIO. Based on the findings of this retrospective study, we recommend considering a greater microkeratome head size > 450 μm or greater when clinically appropriate, to enhance the precision of the stromal cut and potentially decrease the incidence of TIO. This is particularly relevant when the pre-cut donor cornea thickness exceeds 550 μm and the aiming target is an ultra-thin DSAEK graft measuring less than 100 μm. In such cases, both the difference between the pre-cut corneal graft thickness and the post-cut DSAEK lenticule thickness, along with the relationship between dissection thickness and microkeratome head size, are likely to increase, raising the likelihood of TIO occurrence.

This study is retrospective, and one of its limitations is the absence of precise documentation regarding the duration of the microkeratome pass and the pressure applied within the artificial anterior chamber during the DSAEK preparation of the donor corneas included. These variables may have influenced the depth of dissection and, consequently, could have contributed to the formation of TIOs. Additionally, the potential impact of donor corneal edema on pachymetry could not be accurately assessed. In cases where increased pachymetry is due to significant corneal edema, the dehydration that occurs during the stabilization of the artificial chamber could influence the precision of the stromal cut and the subsequent occurrence of TIOs. This study was conducted at a single institution using standardized surgical techniques and postoperative protocols to minimize confounding variables. However, this may limit the generalizability of the findings, as variations in surgical approaches, equipment, and patient demographics at other centers could yield different outcomes. Finally, although donor lens status was included in the analysis, other aspects of donor ocular history and demographics were not comprehensively assessed and may influence graft characteristics and outcomes.

## 5. Conclusions

In conclusion, our study indicates a statistically significant correlation between TIOs and the microkeratome-cutting process used in the preparation of DSAEK corneal grafts, while we propose specific techniques that may help reduce the incidence of TIOs. Specifically, creating an ultra-thin DSAEK lenticule using a smaller microkeratome head with the slow single-pass technique may increase the risk of TIOs. Conversely, larger microkeratome heads can enhance stromal thickness consistency and reduce TIO incidence. However, further research needs to be conducted to evaluate and compare the occurrence and severity of TIOs across various microkeratome DSAEK preparation techniques

## Figures and Tables

**Table 1:** Pre-cut graft thickness, post-cut DSAEK lenticule thickness, and their difference by M-TIO grade.

	M-TIO Grade			
Variable, mean ± SD	0	1	2	3
Pre-cut graft thickness (µm)	502 ± 54	516 ± 61	531 ± 63	552 ± 78
Post-cut DSAEK lenticule thickness (µm)	91 ± 19	88 ± 19	87 ± 16	84 ± 16
Difference between the pre-cut graft thickness and post-cut DSAEK lenticule thickness (µm)	412 ± 57	428 ± 62	444 ± 62	468 ± 78
N	76	224	97	25

**Table 2:** Univariable associations of donor variables with TIO (N = 422 donor corneas).

		Ordinal Logistic Regression Model	
	N = 422	Proportional OR (99% CI)	*p*-Value
Pre-cut Donor Cornea Thickness (µm)			<0.001
		1.35 (1.10, 1.67) per 50 µm	
Post-cut DSAEK lenticule thickness (µm)			0.08
		0.67 (0.36, 1.22) per 50 µm	
Difference Pre-cut graft thickness and the post-cut DSAEK lenticule thickness (µm)			<0.001
		1.38 (1.13, 1.72) per 50 µm	
Microkeratome head (µm)			<0.001
300	115	1.60 (0.26, 6.77)	
350	270	1.98 (0.33, 8.07)	
400	20	11.73 (1.31, 131.24)	
450 or 500	16	1 [ref]	
Dissection thickness vs. microkeratome head (µm)			<0.001
		1.54 (1.22, 1.97) per 50 µm	
Microkeratome			0.51
MORIA 1&2	131	1 [ref]	
MORIA 3&4	124	0.96 (0.50, 1.85)	
MORIA 5&6	112	1.08 (0.56, 2.05)	
MORIA 7&8 (LINEAR MODEL)	52	1.53 (0.63, 4.15)	
Donor lens status			0.67
Phakic	393	1 [ref]	
Pseudophakic	25	1.31 (0.25, 6.30)	
Year			<0.001
2019	43	1 [ref]	
2020	36	1.52 (0.35, 6.48)	
2021	51	1.81 (0.61, 5.62)	
2022	108	3.93 (1.38, 12.01)	
2023	184	1.70 (0.67, 4.42)	

Abbreviations: CI = confidence interval; OR = odds ratio; TIOs = textural interface opacities.

**Table 3:** Multivariable model for predicting TIOs, selected using all possible regressions with BIC (N = 422 donor corneas).

		Ordinal Logistic Regression Model	
	N = 422	Proportional OR (99% CI)	*p*-Value
Difference between pre-cut graft thickness and post-cut DSAEK lenticule thickness (µm)			<0.001
		1.57 (1.22, 2.06) per 50 µm	
Microkeratome head (µm)			<0.001
300	115	6.95 (1.04, 36.60)	
350	270	4.39 (0.76, 19.00)	
400	20	18.86 (2.35, 175.91)	
450 or 500	16	1 [ref]	

Abbreviations: BIC = Bayesian information criterion; CI = confidence interval; OR = odds ratio; TIO = textural interface opacity.

## Data Availability

The data presented in this study are available on request from the corresponding author. The data are not publicly available due to privacy or ethical restrictions.
